# Effect of treated sunflower meal with tannin extracted from pistachio hulls on *in vitro* gas production and ruminal fermentation

**Published:** 2017-09-15

**Authors:** Alireza Jolazadeh, Tahereh Mohammadabadi

**Affiliations:** Department of Animal Science, College of Animal Science and Food Technology, Ramin Agriculture and Natural Resources University of Khuzestan, Mollasani, Iran.

**Keywords:** Fermentation, Gas production, Pistachio hull, Sunflower meal, Tannin

## Abstract

This experiment was conducted to study the effects of various amounts of treated sunflower meal (SFM) with extracted tannins from pistachio hulls on *in vitro* gas production and ruminal fermentation in ruminants. The SFM was treated with pistachio extract concentrate (PEC), which contained 111.40 g kg^-1^ total phenol and 71.30 g kg^-1 ^total tannin per dry matter of extract, at six experimental treatment levels of 0, 7, 14, 21, 28 and 35 g PEC per 100 g SFM on a dry matter basis. *In vitro* gas production, *in vitro* organic matter digestibility (IVOMD), metabolizable energy (ME) and fermentative parameters of samples were measured. The PEC had no effect on fermentation rate, but asymptotic gas production was linearly decreased with increasing dose of extract. All fermentation parameters (*i.e*., apparent degraded substrate, short chain fatty acids, gas yield at 24 hr, partitioning factor at 96 hr, IVOMD, ME and microbial protein production (MP) linearly decreased with increasing PEC treatment. Inclusion of PEC linearly decreased ruminal ammonia nitrogen concentration (NH_3_-N), total protozoa population and pH in the all incubation times. In conclusion, the addition of PEC positively modified some rumen parameters such as NH_3_-N concentration and protozoa population.

## Introduction

When animals are fed high protein diets, it would be interesting to reduce ruminal degradability of crude protein, allowing part of this protein to reach the small intestine, thus avoiding possible losses of amino acids in the rumen due to microbial fermentation since this fermentation process is inefficient and generally presents losses up to 10%, mainly as ammonia nitrogen (NH_3_-N).^1^

Protein degradation in the ruminal environment should provide only the necessary amount of nitrogen to meet the microbial requirements, improving the bacteria growth and consequently nutrient digestion. Protection of dietary protein from ruminal microbial degradation increases the supply of amino acids to the small intestine and loss of nitrogen may be reduced by decreasing protein degradation in the rumen.^2^^,^^3^ Therefore, the efficiency of protein utilization by the animal should be improved.^4^ Sunflower meal (SFM) is a by-product obtained from sunflower oil extraction. The SFM is used as a supplemental protein in ruminant diets and is classified as a highly degradable protein source in the rumen.^5^ Feeding SFM is often limited by high ruminal degradability,^6^ but it would be possible to improve its nutritive value using diets rich in tannins for decreasing protein degradability, such as pistachio extract concentrate (PEC) extracted from pistachio hull by-products.

 There are some methods to decrease protein degradation in rumen such as using tannins. Tannins have been shown to decrease ruminal degradation of crude protein (CP) and to increase the amount of CP that reaches the abomasum and small intestine.^7^ However, Dentinho *et al*. reported decreased effective rumen degradability of soybean meal (SBM) protein treated with tannin extracts from *Citrus Ladanifer* and decreased *in vitro *intestinal digestibility of protein at low phenolic doses (12.50, 25 and 50 g total phenol per kg).^8^ When using SBM treated with 10 to 250 g of quebracho tannins per kg, Frutos *et al*. also observed decreased *in vitro* intestinal digestibility of protein at the greatest treatment rate.^9^ It has been demonstrated that feeding SBM-treated PEC increases average daily gain and feed efficiency in Holstein bulls.^10^ Further research is needed to determine if tannin treatment of dietary protein improves its digestibility.

High amounts of pistachio by-products have been produced in Iran at an average rate of about 310,000 metric tons annually.^11^ Pistachio by-products are produced during de-hulling of pistachio nuts after harvesting and contain high concentrations of phenolic compounds and tannins, which can affect utilization of their nutrients by animals.^12^ Water has effectively been used as a solvent during extraction to decrease cost and make PEC and economically feasible treatment for animal diets.^10^

This experiment was conducted to assess the effects of five levels of PEC used in treating SFM extracted by water, on *in vitro* ruminal fermentation of SFM protein. We hypothesized that PEC treatment would improve digestive utilization of SFM protein in ruminant diets.

## Materials and Methods

**Animal care. **All animal management and sampling procedures were conducted according to The Care and Use of Agricultural Animals in Research and Teaching guidelines.^13^ All procedures and guidelines involving animals were approved by the Animal Experiment Committee at Ramin Agriculture and Natural Resource University, Molasani, Khuzestan, Iran.

**Pistachio hull, tannin extraction and treatments**. Pistachio hulls (Fandoghi variety) were obtained from the pistachio processing factory related to the holy shrine of Lady Fatimah Masoumeh, Qom, Iran, during the summer of 2014 and were sun-dried. A two-step extraction process was performed according to procedure by Jolazadeh *et al*. to obtain PEC, which contained 111.40 g per kg total phenol and 71.30 g per kg total tannin on a dry matter (DM) basis.^10^ Chemical composition of PEC and SFM treated with various levels of PEC are shown in Table 1. Samples of SFM and PEC were analyzed in triplicate for DM, ash and CP according to AOAC procedure numbers of 930.15, 94.05 and 984.13, respectively.^14^ Analysis of phenolic compounds was conducted in three replicates as described by Makkar.^15^ Total phenol was determined by using Folin-Ciocalteu’s reagents and the concentration was measured as tannic acid equivalent using tannic acid (Merck, Darmstadt, Germany) as a standard. Total tannins were measured as described by Makkar.^15^

A total amount of 100 g dried SFM was ground through a 1 mm screen for each treatment. Then, PEC was added to ground SFM to achieve ratios of 0, 70, 140, 210, 280 or 350 mg PEC per g SFM. Treated SFM was then air-dried for 12 hr to reach DM content of about 900 g per kg.

**Animals and rumen liquor sampling.** Two fistulated rumen of Holstein cows with body weight of about 620 ± 12 kg were used as rumen liquor donors. The cows were fed twice daily with a total mixed ration diet containing 40:60 concentrate: forage ratio and each cow received 140 g PEC per day for microbial adaptation to PEC tannins. Rumen liquor was sampled before the morning feeding at 08:30 AM from the two cows and placed immediately in warm (39 ˚C) insulated flasks under anaerobic conditions. In the laboratory, samples were pooled in equal proportions and strained through eight layers of cheesecloth under anaerobic conditions and used immediately.

**Table 1.** Chemical composition (means ± SD) of pistachio extract concentrate (PEC) and sunflower meal (SFM) treated with various levels of PEC, as dry matter basis

**Treatments** *	**Chemical composition, g per kg dry matter**					
	**Dry matter**	**Crude protein**	**Organic matter**	**Ash**	**Total phenolic**	**Total tannin**
**PEC**	850.00 ± 2.50	153.00 ± 0.80	777.00 ± 1.70	224.00 ± 1.70	112.00 ± 0.40	71.30 ± 0.30
**SFM-0**	922.00 ± 2.10	283.00 ± 0.30	921.00 ± 0.80	78.20 ± 0.40	0.00	0.00
**SFM-7**	917.00 ± 2.30	274.00 ± 0.40	911.00 ± 0.40	88.30 ± 0.40	78.40 ± 0.20	49.90 ± 0.40
**SFM-14**	912.00 ± 2.10	265.00 ± 0.30	901.00 ± 1.10	98.50 ± 0.80	156.00 ± 0.10	99.80 ± 0.60
**SFM-21**	907.00 ± 2.40	255.00 ± 0.60	891.00 ± 0.40	108.00 ± 0.70	235.00 ± 0.40	149.00 ± 0.50
**SFM-28**	901.00 ± 2.20	246.00 ± 0.10	881.00 ± 1.20	119.00 ± 1.20	313.00 ± 0.70	199.00 ± 0.20
**SFM-35**	897.00 ± 2.20	237.00 ± 0.50	871.00 ± 0.30	129.00 ± 0.70	392.00 ± 0.20	249.00 ± 0.40

* SFM-0 to SFM-35 was treatments supplemented with 0, 7, 14, 21, 28 and 35 g PEC per 100 g of SFM, respectively.

***In vitro*** **gas production. **Gas production kinetics for PEC-treated SFM was determined as described by Menke and Steingass.^16^ Prepared rumen fluid was added to the anaerobic mineral buffer solution (1:2 v/v). Buffer medium composition per liter was NaHCO_3_, 70.00 g; NH_4_HCO_3_, 4.00 g; Na_2_HPO_4_, 5.70 g; KH_2_PO_4_, 6.20 g; MgSO_4_·7H_2_O, 0.60 g; Na_2_S, 0.52 g; CaCl_2_·H_2_O, 13.20 g; MnCl_2_·4H_2_O, 10.00 g; CoCl_2_·6H_2_O, 1.00 g; resazurin sodium salt 0.01 g and 60 mL freshly prepared reduction solution containing 580 mg Na_2_S·9H_2_O, 3.70 mL and 1 mol L^-1^ NaOH. The mixture was kept stirred under CO_2_ flushing at 39 ˚C using a magnetic stirrer fitted on a hot plate. Effect of tannins on protecting of SFM was assessed by incubating approximately 200 mg experimental sample (1.00 mm screen, eight replicates) with 30 mL of rumen buffer mixture in 100 mL glass syringes based on Menke and Steingass procedures.^16^ Analyses were completed in eight replicates with readings of gas production at 0, 2, 4, 6, 8, 12, 24, 48, 72, 96 and 120 hr of incubation. Total gas values were corrected for the blank. 

**Calculations. **After 96 hr of incubation with rumen buffer mixture, the culture fluid of each syringe was used for determination of NH3-N concentration using distillation method using an autoanalyzer (Kjeltec 2300; Foss Tecator AB, Hoganas, Sweden). The pH was determined immediately using a portable pH meter (Model A102-003; Sentron, Roden, The Netherlands). Protozoa were counted using a Burker counting chamber (Blau Brand, Wertheim, Germany) according to the method described by Veira *et al*.^17^ Cumulative gas production data were fitted to the exponential equation as:

*Y = b (1 − e* ^− ct^ *)*

where, *b* is gas production from the fermentable fraction (mL), *c* is the gas production rate constant (mL per hr), *t* is the incubation time (hr) and *Y* is gas produced in time *t*. 

Short chain fatty acids (SCFA) were determined by the equation reported by Getachew *et al*.^18^ The SCFA (mmol per 200 mg DM) = 0.0222 GP − 0.00425, GP was the net gas production (mL per 200 mg DM) after 24 hr incubation. The DM degradability at 24 hr of incubation (apparent degraded substrate, ADS; mg per g DM) was calculated as the difference between DM content of substrate before incubation and its undegradable DM after incubation.^19^

*ADS = DM content of substrate before incubation – undegradable DM after incubation metabolizable energy (ME, MJ kg* ^-1^ *DM)*

and *in vitro* organic matter disappearance (IVOMD) were estimated according to Menke and Steingass as: 

*OMD (g kg* ^-1^ *OM) = 148.80 + 8.89 GAS + 4.50 CP + 0. 651 XA*

and 

*ME (MJ kg per DM) = 2.20 + 0.136 GAS + 0.057 CP*

where, OMD is organic matter disappearance, ME is metabolizable energy, CP is crude protein in g per 100 g DM, XA is ash in g per 100 DM and GAS is the net gas production (mL) for 200 mg of sample.^16^ Gas yields (GY_24_) were calculated as the volume of gas produced after 24 hr (mL gas per g ADS) of incubation divided by the amount of ADS (g) as: 

*GY*_24_ *= mL gas per g DM per g of ADS*

Microbial protein production (MP) was calculated as: 

*MP (mg per g of DM) = mg ADS − (mL gas × 2.20 mg mL* ^-1^ *)*

Where, the 2.20 mg mL^-1^ is a stoichiometric factor that expresses mg of C, H and O required for the volatile fatty acids’ gas associated with production of 1 mL of gas.^20^ The ratio of organic matter truly degraded (mg) to produced gas (mL) after 24 hr of incubation was used to estimate the partitioning factor (PF).^19^

**Statistical analysis. **All data were subjected to one-way analysis of variance in which effects of treatments (i.e., different levels of PEC) were partitioned into linear and quadratic components by orthogonal polynomials. All analyses used procedures SAS (version 9.0; SAS Institute, Cary, USA): 

*Yij = μ+ Si+ eij*

where, *Y*_ij_ is the general observation, *μ*_ij_ is the general mean, *S*_i_ is the *i*th effect of extracts on the observed parameters and *e*_ij_ is the standard error term. Means were tested using Duncan’s test at *p *< 0.05.

## Results

*In vitro* gas production parameters and gas volume accumulated after different hours of incubation are presented in. At all incubation times, gas production was visibly linearly decreased due to incorporation of tannin extract (Table 2). The PEC had no effect on fermentation rate (c), but asymptotic gas production (b) was linearly decreased (*p* < 0.01) with increasing dose of extract. Ruminal fermentation parameters of SFM treated with various levels of PEC are shown in Table 3. All fermentation parameters (i.e., ADS, SCFA, GY_24_, PF_96_, IVOMD, ME and MP) linearly decreased (*p* < 0.05) with increasing PEC treatment. Inclusion of PEC linearly decreased (*p *< 0.01) ruminal ammonia nitrogen concentration, total protozoa population (*p *< 0.01) and pH (*p* < 0.01) in all the incubation times (Table 4). 

**Table 2:** *In vitro* gas production parameters and gas volume accumulated after different hours of incubation in untreated and treated sunflower meal (SFM

**Parameters**	**Levels of treated SFM** *						**SEM**	***p*** **-value**	
	**SFM-0**	**SFM-7**	**SFM-14**	**SFM-21**	**SFM-28**	**SFM-35**		**Linear**	**Quadratic**
***In vitro gas production (mL per g of DM)***									
**Gas 12**	9.63^a^	8.17^a^	5.38^b^	3.97^bc^	1.12d	2.63^cd^	0.89	< 0.01	0.08
**Gas 24**	23.38^a^	22.87^a^	18.80^a^	10.63^b^	11.05b	7.65^b^	1.80	< 0.01	0.15
**Gas 48**	35.85^a^	36.62^a^	33.33^a^	24.95^b^	24.12b	20.63^b^	2.07	< 0.01	0.38
**Gas 72**	41.95^a^	43.58^a^	40.63^a^	33.63^b^	31.85b	29.75^b^	1.72	< 0.01	0.35
**Gas 96**	44.97^a^	47.20^a^	44.33^a^	38.63^b^	36.45b	35.72^b^	1.54	< 0.01	0.39
**Gas 120**	45.30^a^	47.43^a^	45.45^a^	38.78^b^	37.67b	36.43^b^	1.57	< 0.01	0.83
***Gas production parameters***									
**Asymptotic gas production** **(mL)**	61.75^a^	58.47^a^	58.96^a^	56.19^ab^	51.18^b^	53.23^b^	2.06	< 0.01	0.42
**Fermentation rate (hr)**	0.022	0.019	0.013	0.024	0.23	0.14	0.005	0.67	0.73

* SFM-0 to SFM-35 was treatments supplemented with 0, 7, 14, 21, 28 and 35 g PEC per 100 g of SFM, respectively.

abcd different superscript letters in each row indicate significant differences at *p* < 0.05.

**Table 3:** *In vitro* rumen fermentation parameters of sunflower meal (SFM) treated with various levels of pistachio extract concentrate

**Parameters**	**Levels of treated SFM** *						**SEM**	***p*** **-value**	
	**SFM-0**	**SFM-7**	**SFM-14**	**SFM-21**	**SFM-28**	**SFM-35**		**Linear**	**Quadratic**
**ADS (mg per g of DM)**	98.50^a^	97.00^ab^	95.60^ab^	94.10^ab^	92.00^ab^	90.50^b^	1.59	< 0.01	0.78
**PF**_96_ ** (mg ADS per mL gas)**	4.35b	4.09^b^	4.20b	4.86^a^	5.04^a^	4.97^a^	0.09	< 0.01	0.19
**GY**_24_ ** (mL gas per g ADS)**	229.90^a^	240.90^a^	237.70^a^	205.60^b^	198.30^b^	201.00^b^	3.48	< 0.01	0.12
**MP (mg per g of DM)**	48.67^ab^	44.84^b^	45.61^ab^	51.58^a^	51.91^a^	50.51^ab^	1.59	0.05	0.56
**SCFA (** **mmol per g of DM)**	1.00^a^	1.05^a^	1.00^a^	0.85^b^	0.83^b^	0.80^b^	0.38	< 0.01	0.44
**ME (** **MJ per kg of DM)**	9.96^a^	10.19^a^	9.87^a^	8.86^b^	8.70^b^	8.48^b^	0.23	< 0.01	0.44
**IVOMD (** **g per kg)**	68.04^a^	69.58^a^	67.49^a^	60.91^b^	59.90^b^	58.41^b^	1.46	< 0.01	0.44

ADS: Apparent degraded substrate; IVOMD: *In vitro* organic matter disappearance; PF_96_: Partitioning factor at 96 hr of incubation; MP: Microbial protein production; GY_24_: Gas yield at 24 hr; SCFA: Short chain fatty acids; ME: Metabolizable energy; SEM: Standard error of the mean.

* SFM-0 to SFM-35 was treatments supplemented with 0, 7, 14, 21, 28 and 35 g pistachio extract concentrate per 100 g of SFM, respectively.

ab different superscript letters in each row indicate significant differences at *p* < 0.05.

**Table 4:** The effect of pistachio extract concentrate (PEC)-treated sunflower meal (SFM) on total protozoa numbers, pH and ammonia concentration at 24 hr incubation time

**Parameters**	**Levels of treated SFM**						**SEM**	***p*** **-value**	
	**SFM-0**	**SFM-7**	**SFM-14**	**SFM-21**	**SFM-28**	**SFM-35**		**Linear**	**Quadratic**
**pH**	6.82^a^	6.79^ab^	6.74^bc^	6.76^bc^	6.74^bc^	6.71^c^	0.02	< 0.01	0.56
**Protozoa (10** ^4^ ** mL** ^-1^ **)**	19.83^a^	19.58^a^	16.66^b^	14.83^b^	9.50^c^	9.00^dc^	0.85	< 0.01	0.17
**NH**_3_ **-N (mg dL** ^-1^ **)**	16.78^a^	16.35^a^	14.14^b^	12.81^c^	10.55^d^	9.46^e^	0.32	< 0.01	0.07

* SFM-0 to SFM-35 was treatments supplemented with 0, 7, 14, 21, 28 and 35 g PEC per 100 g of SFM, respectively.

**abcde:** different superscript letters in each row indicate significant differences at *p* < 0.05.

## Discussion

There is limited information in the literature on the use of pistachio hull extract. Treatment of SFM with PEC did not affect c, but adding PEC linearly decreased b. 

It has been reported that tannin decreases cumulative gas production, probably by formation of tannin–macromolecule complexes which inhibit microbial enzymes and/or nutrient utilization by ruminal anaerobes.^21^^,^^22^ Similar results were obtained by El-Waziry *et al*., who found that adding tannic acid to SBM decreases gas production.^23^ Getachew *et al*. reported that tannic and gallic acids reduce the rate of fermentation *in vitro*.^24^ The researchers concluded that treatment with tannic acid decreases the degradation (b and c) of SBM protein.^22^ Reductions in digestibility have been observed *in vivo* only when forages containing over 5.00% DM condensed tannin are fed.^25^ It has been shown that tannins significantly depress gas production, probably through hampering rumen microorganisms.^26^

In the present study, ADS, IVOMD, gas production at 24 hr (GP_24_), PF_96_, MP and ME were decreased with increased rates of PEC treating SFM due to decreased rumen degradability and formation of the complexes between tannins and dietary protein and carbohydrates as well as reducing rumen microbial proteolysis and cellulolytic enzyme activities. It has been reported that SFM contains 18.04% non-fiber carbohydrates, 42.36% neutral detergent fiber, 26.94% acid detergent fiber, 15.42% hemicellulose, 8.57% cellulose and 6.85% lignin (% of Dry Matter) as carbohydrates concentration.^27^ It has been demonstrated that diet supplementation with pomegranate-peel extracts decreases ADS, but has no effect on IVOMD, ME, PF_24_, GY_24_ and MP under *in vitro* condition in the sheep.^28^ In contrast, another report has noted that addition of different doses of *Leucaena leucocephala* and *Salix babylonica* extracts (0.60, 1.20, 1.80 mL extract per g of DM) containing a low total phenolic concentration (<5% of DM) and saponins increases gas volume, GP_24_, ADS and MP versus the control.^29^

Values of SCFA and MP (mg per g of DM) in different treatments are shown in Table 3. These results are in accordance with findings of EL-Waziry *et al*. that reported the volatile fatty acid concentrations are significantly decreased when SBM is treated by tannic acid.^23^ There is a positive correlation between SCFA and gas production and gas production is a good predictor for the production of volatile fatty acid, which is positively related to microbial mass production.^16^^,^^30^

In this study, rumen protozoal numbers were decreased with increased rates of PEC treatment, as shown in Table 4. The antiprotozoal effect of PEC was most likely due to the phenolic structure of active compounds. Phenolic structures may disrupt protozoal membranes, inactivate protozoal enzymes and deprive protozoa of substrates and metal ions which are essential for cell metabolism.^31^ Results from our study confirm findings by Baah *et al*., who found that dietary supplementation of quebracho tannin tends to cause a decrease in the total protozoal numbers in ruminal fluid of heifers.^32^

In all experimental groups in the present study, the mean ruminal pH values were within the normal physiological range of 6.10 to 6.80 reported by Van Soest.^33^ Tannins have been reported to cause ruminal pH decrease and/or increase, however they may have no effect on it.^34^^-^^36^

 The reduction in ammonia concentration suggests that inclusion of tannins probably reduces proteolysis, degradation of peptides and deamination of amino acids.^33^ This is in consistent with El-Waziry *et al*. findings who showed that adding tannins to SBM decreases ammonia production *in vitro*.^22^ Moreover, formation of tannin–protein complexes in the rumen likely caused a decrease in ruminal protein degradation and lowered concentration of ruminal ammonia nitrogen *in vitro*. These results are in accordance with findings of West et al.^37^ Decreased concentration of ammonia nitrogen in the rumen is typical when protozoal numbers are decreased.^38^

In conclusion, treating SFM with PEC decreased the concentration of ammonia and total protozoal population in the rumen fluid. *In vitro* gas production parameters were decreased with increasing levels of the PEC extract in the treatment. *In vivo* studies must be conducted to validate the *in vitro* results.
